# Co-circulation of Usutu virus and West Nile virus in a reed bed ecosystem

**DOI:** 10.1186/s13071-015-1139-0

**Published:** 2015-10-12

**Authors:** Ivo Rudolf, Tamás Bakonyi, Oldřich Šebesta, Jan Mendel, Juraj Peško, Lenka Betášová, Hana Blažejová, Kristýna Venclíková, Petra Straková, Norbert Nowotny, Zdenek Hubálek

**Affiliations:** Institute of Vertebrate Biology, v.v.i., Academy of Sciences, Květná 8, 60365 Brno, Czech Republic; Department of Microbiology and Infectious Diseases, Faculty of Veterinary Science, Szent István University, Budapest, Hungary; Viral Zoonoses, Emerging and Vector-Borne Infections Group, Institute of Virology, University of Veterinary Medicine, Vienna, Austria; Department of Experimental Biology, Faculty of Science, Masaryk University, Brno, Czech Republic; Department of Microbiology and Immunology, College of Medicine and Health Sciences, Sultan Qaboos University, Muscat, Oman

**Keywords:** *Culex modestus*, Usutu virus, West Nile virus, Flavivirus, Arbovirus, Surveillance, Mosquitoes

## Abstract

**Background:**

Mosquito-borne flaviviruses are a major public health threat in many countries worldwide. In Central Europe, West Nile virus (WNV) and Usutu virus (USUV), both belonging to the Japanese encephalitis virus group (*Flaviviridae*) have emerged in the last decennium. Surveillance of mosquito vectors for arboviruses is a sensitive tool to evaluate virus circulation and consequently to estimate the public health risk.

**Methods:**

Mosquitoes (*Culicidae*) were collected at South-Moravian (Czech Republic) fishponds between 2010 and 2014. A total of 61,770 female *Culex modestus* Ficalbi mosquitoes, pooled to 1,243 samples, were examined for flaviviruses by RT-PCR.

**Results:**

One pool proved positive for USUV RNA. Phylogenetic analysis demonstrated that this Czech USUV strain is closely related to Austrian and other Central European strains of the virus. In addition, nine strains of WNV lineage 2 were detected in *Cx. modestus* collected in the same reed bed ecosystem.

**Conclusions:**

This is the first detection of USUV in *Cx. modestus.* The results indicate that USUV and WNV may co-circulate in a sylvatic cycle in the same habitat, characterised by the presence of water birds and *Cx. modestus* mosquitoes, serving as hosts and vectors, respectively, for both viruses.

## Background

Usutu virus (USUV) is a mosquito-borne virus (Japanese encephalitis group, genus *Flavivirus*; family *Flaviviridae*) that was originally isolated in Africa. In or before 1996, the virus was introduced to Europe [1]. It circulates in nature between birds (as amplifying hosts) and bird-feeding mosquitoes, principally *Culex* spp., as vectors. USUV is taxonomically and ecologically very similar to West Nile virus (WNV) [2, 3]. Contrary to WNV, USUV has rarely caused human disease – only in immunocompromised persons [4]. However, USUV antibodies were recently reported in three patients with neuroinvasive disease in Croatia [5].

In the Czech Republic, two strains of USUV were isolated from dead blackbirds (*Turdus merula*) in Brno, 2011 and 2012 [6]. In addition, specific neutralizing antibodies against USUV were found in common coots (*Fulica atra*) in Moravia [7, 8].

Neutralizing antibodies against WNV were rarely found in the local human population, but five cases of West Nile fever in humans were reported after heavy floods in 1997 [9]. More frequently WNV antibodies occur in apparently healthy wild birds in this region [7]. Three identical strains of WNV (proposed genomic lineage 3: Rabensburg) were isolated from *Culex pipiens* and *Aedes rossicus* mosquitoes in 1997, 1999 and 2006 [10]. In a previous study, we reported four strains of lineage 2 WNV from *Culex modestus* mosquitoes collected in reed beds at South-Moravian fishponds (Czech Republic) during August 2013 [11].

Within the scope of the joint European EDENext project we extended our virological surveillance of local *Cx. modestus* mosquitoes for pathogenic flaviviruses, including USUV.

## Methods

### Study sites

**Fig. 1:**
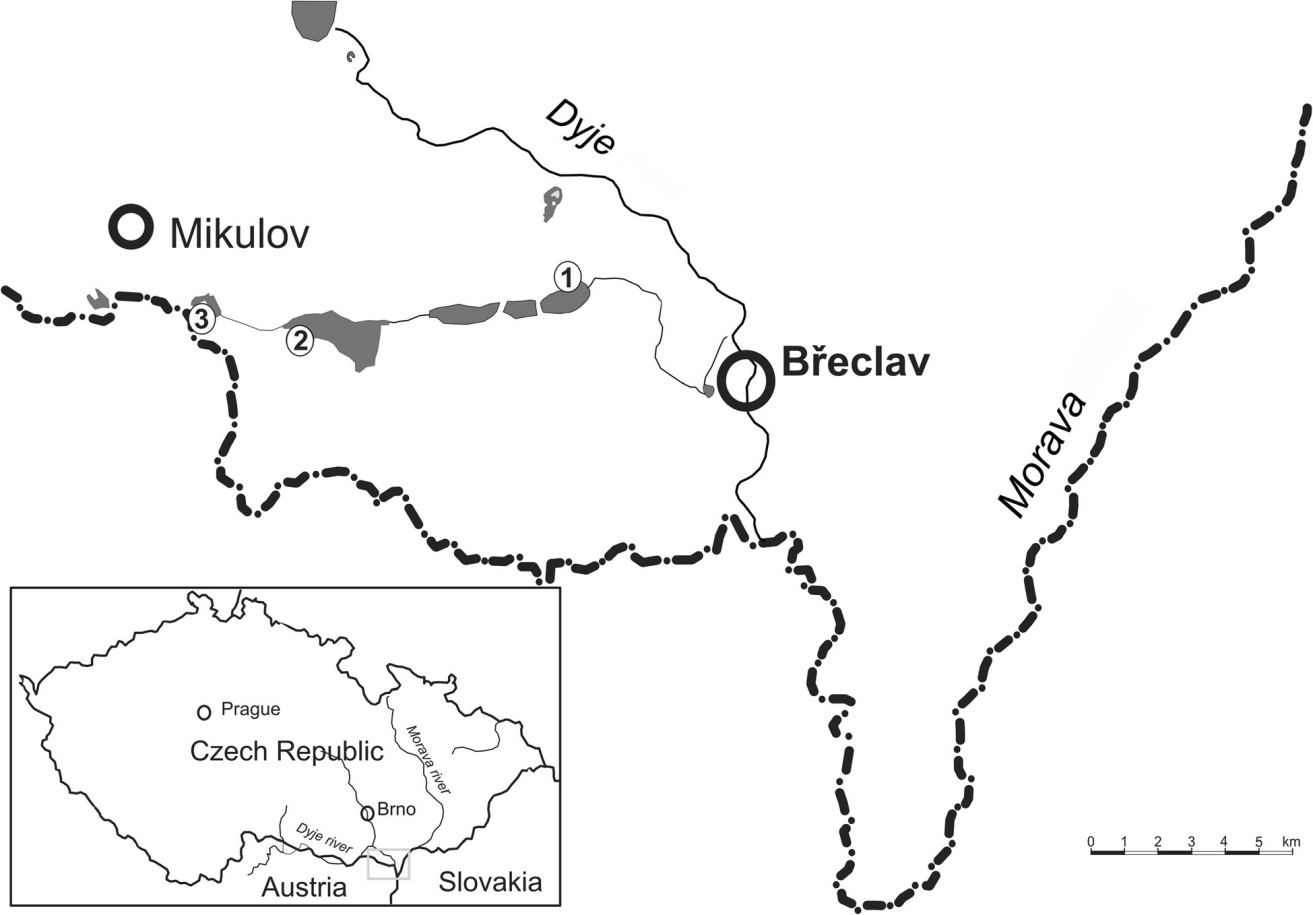
Study sites in south Moravia: 1, fishpond “Mlýnský”; 2, fishpond “Nesyt”; 3, fishpond “Nový”

Female mosquitoes were collected using CDC minilight CO_2_-baited traps (EVS CO_2_ Mosquito Trap, BioQuip Products Inc., United States) at three study sites (fishponds “Nesyt” and “Nový” near Sedlec, and “Mlýnský” near Lednice: 48°47′ N and 16°42′ − 16°49′ E; 175–177 m a.s.l.) in the district of Břeclav, South Moravia, Czech Republic, as described in a preceding paper [11], during July and August from 2010 to 2014. All study sites are characterised by reed bed ecosystem (*Phragmites communis* alliance) situated at the littoral zone of the fishponds (Fig. 1). Thirty species of birds have been recorded breeding in the reed bed, and an additional 54 wild wetland and terrestrial bird species visit this ecosystem during seasonal movements [12]. A characteristic mosquito species for this ecosystem in this part of Moravia is *Cx. modestus*. From an epidemiological point of view it is noteworthy that all study sites represent favourite recreational areas during the summer season.

### Mosquito processing, RNA extraction, PCR and sequencing

Caught insects were transported to the laboratory in cooled flasks, and stored at −65 °C until examination. Mosquitoes were determined on a chilled table under a stereomicroscope according to an entomological key [13], and monospecific pools consisting of up to 50 female *Cx. modestus* (other species were not tested in this study) were homogenized in 1.5 ml of cooled phosphate-buffered saline pH 7.4 supplemented with 0.4 % bovine serum albumin (Sigma) and antibiotics (PBS-BSA), and centrifuged.

RNA was extracted from 140 μl mosquito homogenates using the QIAamp Viral RNA Mini Kit (Qiagen, Hilden, Germany) according to the manufacturer’s instructions. Generic oligonucleotide primers targeting the NS5 region of flaviviruses were used for screening purposes [14]. Samples positive by the above pan-flavivirus PCR were subjected to USUV [15, 16] as well as WNV [11] -specific RT-PCR assays for amplification of overlapping genome regions. Amplification products were directly sequenced (Microsynth, Balgach, Switzerland); the sequences were aligned and compiled, and identified by BLAST search against GenBank database.

### Phylogenetic analyses

Phylogenetic and molecular evolutionary analyses of concatenated sequences were conducted using neighbor-joining, maximum likelihood, minimum evolution, UPGMA and maximum parsimony algorithms (MEGA version 6, with 1000 replicates for bootstrap testing).

### Virus isolation

The original mosquito homogenates of PCR-positive samples were inoculated intracerebrally (i.c., 20 μl) into specific pathogen free suckling ICR mice (SM). Brains of dead animals were homogenized in PBS-BSA, centrifuged, and passaged (i.c.) in a new batch of SM. Bacterial sterility of the suspensions was checked in meat-peptone and thioglycollate broths incubated at 37 °C [10]. All experiments with laboratory mice were conducted in accordance with the Czech Animal Protection Act no. 246/1992, and the protocols were approved by the Institutional and Central Care and Use Committees at the Academy of Sciences of the Czech Republic in Prague and by the Veterinary Service in Brno. The facility is accredited by the Czech National Committee on Care and Use of Laboratory Animals (6630/2008-10001).

## Results and discussion

A total of 61,770 female *Cx. modestus* mosquitoes in 1,243 pools (including 32,500 individuals in 650 pools, collected in the same place and evaluated in 2014 [11]) were examined for flaviviruses by RT-PCR (Table 1). USUV RNA was detected in one pool (#13-662) of *Cx. modestus* collected at Mlýnský fishpond on 7 August 2013; the overall minimum prevalence rate of USUV in *Cx. modestus* was therefore 0.016 per 1,000 mosquitoes. When the mosquito homogenate #13-662 was inoculated into 13 SM, two of them were missing on day 4 p.i. (most probably cannibalized by the mother after they became ill or died). Repeated inoculation of another litter of 12 SM with the same homogenate was negative, all SM survived.

**Table 1:** Numbers of female *Culex modestus* mosquitoes examined for flaviviruses in individual years and at three study sites

Fishpond	2010	2011	2012	2013	2014	*Total*
Nesyt	11 (1)	1,304 (27)	2,649 (54)	8,400 (168)	100 (2)	*12,464 (252)*
Nový	0	0	0	10,835 (217)	206 (4)	*11,041 (221)*
Mlýnský	5,450 (109)	533 (11)	4,079 (82)	22,050 (441)	6,153 (127)	*38,265 (770)*
*Total*	*5,461 (110)*	*1,837 (38)*	*6,728 (136)*	*41,285 (826)*	*6,459 (133)*	*61,770 (1243)*

Number of pools is shown in parentheses

**Fig. 2:**
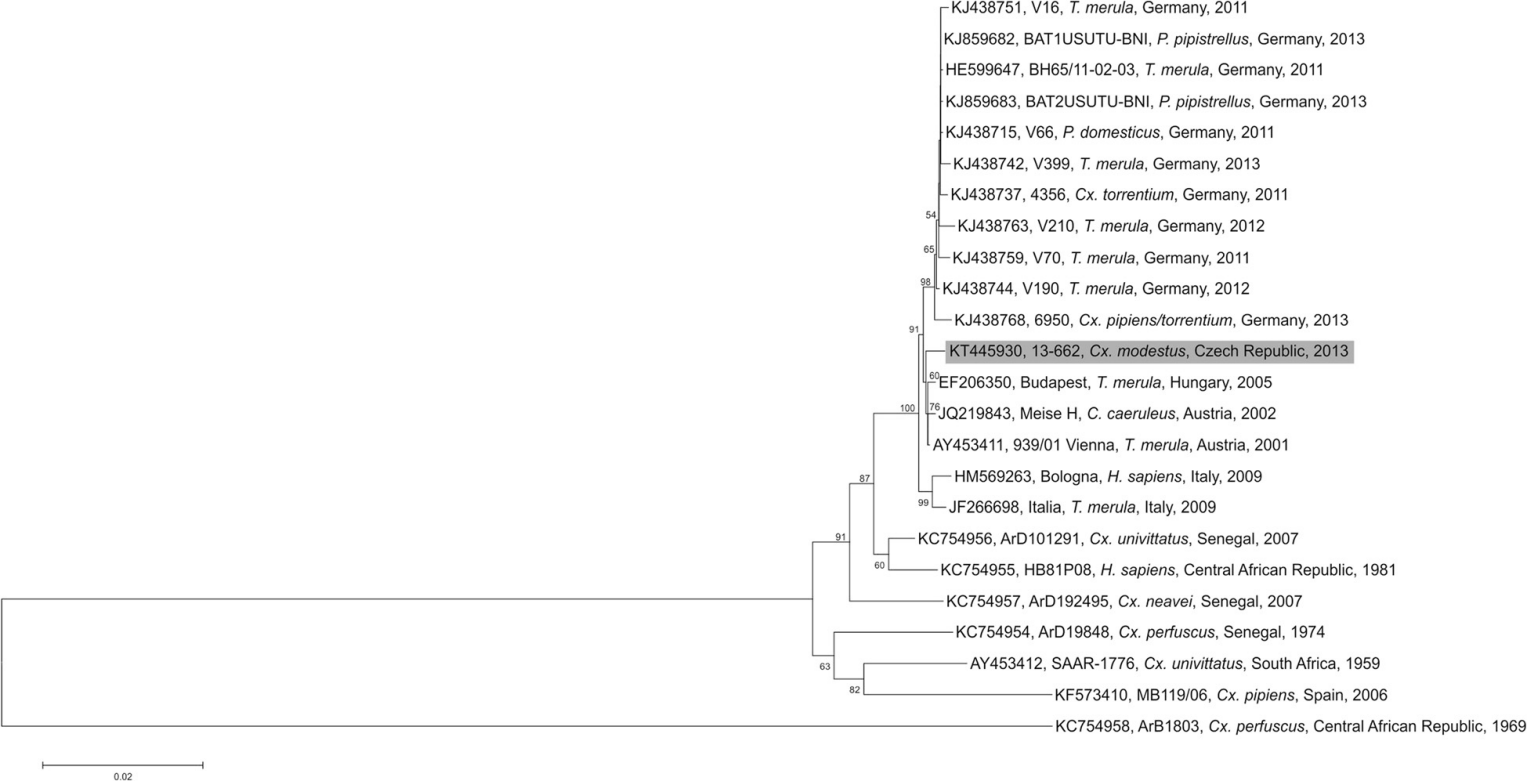
Phylogram demonstrating the genomic relationships among Usutu viruses based on concatenated nucleotide sequences. Partial coding regions were E, NS1, NS2a, NS2b, NS3, NS4a, NS4b and NS5 proteins. Sequences are labelled by codes containing the GenBank accession number, the name of the virus strain, host (*T. merula*: *Turdus merula* [Blackbird], *P. pipistrellus*: *Pipistrellus pipistrellus* [Common pipistrelle], *P. domesticus*: *Passer domesticus* [House sparrow], *Cx.*: *Culex*, *C. caeruleus*: *Cyanistes caeruleus* [Blue tit], *H. sapiens*: *Homo sapiens* [Human]), country of origin, and year of sample collection. The Czech sequence described in this paper is highlighted with grey background. Bootstrap values ≥ 500 (50 %) are displayed. Bar on the left represents the genetic distance

A total of 4218 nucleotides in five genome regions (corresponding to 38 % of the genome) of the USUV-positive pool #13-662 was determined. It revealed 12 substitutions when compared to the complete genome sequence of the USUV Vienna strain from 2001 ([2, 15]; GenBank: AY453411), thus indicating a high (99.7 %) nucleotide identity rate: genome region nt 1610–2980 (E–NS1; 1371 nt): 1 substitution; genome region 3021–3685 (NS1–NS2a; 665 nt): 2 substitutions; genome region 4444–5615 (NS2b–NS3; 1172 nt): 6 substitutions; genome region 6582–6907 (NS4a–2 K; 326 nt): no substitution; genome region 7351–8034 (NS4b–NS5; 684 nt): 3 substitutions. Nucleotide sequences were deposited in GenBank database under accession number KT445930. The phylogenetic relationship of the Czech *Cx. modestus*-derived USUV strain with other USUV strains is displayed in Fig. 2. Neighbor-joining, maximum likelihood, minimum evolution, UPGMA and maximum parsimony algorithms revealed almost identical trees; the phylogeny shown in Fig. 2 is based on the neighbor-joining algorithm. The Czech USUV clustered together with Austrian and Hungarian viruses detected between 2001 and 2005 [16]. However, recently identified USUV strains from Germany and Italy formed separate branches.

Also in South Moravia (Czech Republic), USUV strains had been previously isolated, namely from blackbirds, which were found dead in 2011 and 2012, respectively [6]. The 2011 isolate was sequenced in two regions: partial E + NS1 (GenBank: JX236666) and partial NS5-5′UTR (GenBank: JX236667). In the E + NS1 region there is a 1371 nt overlap (nts 1610–2980 referring to the USUV sequence AY453411) between the 2011 blackbird-derived USUV sequence [6] and the 2013 *Cx. modestus*-derived sequences (this paper), and in the E protein coding region of this overlap the two genomes differ in (only) three nucleotides at positions 1872 (T *vs.* C), 2322 (C *vs.* T), and 2419 (C *vs.* T), resulting in a 99.78 % identity between the two Czech USUV strains in this genomic region; none of the substitutions lead to putative amino acid changes.

Furthermore, WNV genomic lineage 2 (WNV-2) was detected in nine pools of *Cx. modestus* mosquitoes collected in August 2013: #13-104 (collected at Nový fishpond); #13-329 (coll. at Nesyt fishpond); #13-479 (coll. at Mlýnský fishpond); #13-502 (coll. at Mlýnský fishpond) (these four records were reported in a previous study: [11]); #13-670 (coll. at Mlýnský fishpond); #13-743 (coll. at Nesyt fishpond); #13-853 (coll. at Mlýnský fishpond); #13-859 (coll. at Nesyt fishpond); #13-862 (coll. at Nesyt fishpond); the overall minimum prevalence rate of WNV in *Cx. modestus* was therefore 0.146 per 1,000 mosquitoes, about ten times higher than that for USUV. All WNV RNA positive original mosquito homogenates were then inoculated into SM. While the homogenates #13-329, #13-670, #13-743, and #13-853 did not kill any mice, the five others did: #13-104 killed 6 of 11 inoculated SM within 7–8 days post inoculation (DPI), average survival time (AST) of SM was 7.7 days; #13-479 killed 8 of 9 inoculated SM (6–7 DPI; AST 6.1 d); #13-502 killed specifically 7 of 10 SM (6–8 DPI; AST 6.4 d); #13-859 killed 5 of 11 SM (6–7 DPI; AST 6.4 d); and #13-862 killed all 11 inoculated SM (6–7 DPI; AST 6.7 d).

To the best of the authors’ knowledge, this is the first detection of USUV in *Cx. modestus.* It indicates that USUV may co-circulate with WNV in certain habitats – this phenomenon was demonstrated previously in northern Italy, where the principal mosquito vector of USUV (and WNV as well) is *Cx. pipiens* [3, 17–21]. A comprehensive review on the co-circulation of the two arboviruses in Europe has recently been written [22]. Contrary to northern Italy, where USUV occurs in *Culex* mosquitoes much more frequently than WNV, reverse proportion was found in South Moravia in this study.

Interestingly, both viruses (USUV, WNV) were detected in South Moravia in 2013, but not in the years 2010, 2011, 2012 and 2014. This result could be affected by the number of *Cx. modestus* mosquitoes examined in individual years, which was much higher in 2013 than in the other years (Table 1). Moreover, mosquitoes were not collected in August 2014 (only in July).

Our previous finding that the common coot (*Fulica atra*) relatively often reveals specific antibodies to USUV [7, 8] might indicate a specific role of this avian species in the circulation of USUV in wetlands.

## Conclusions

This is the first detection of USUV in *Cx. modestus.* The results indicate that USUV and WNV may co-circulate in a sylvatic cycle in the same habitat, characterised by the presence of water birds and *Cx. modestus* mosquitoes, serving as hosts and vectors, respectively, for both viruses. The present finding suggests that USUV (similar to WNV) may circulate in two types of ecosystems: (i) sylvatic cycle between *Cx. pipiens/ Cx. modestus* and water birds - such as coots, based on a previous serosurvey study [7]; (ii) urban cycle involving *Cx. pipiens* and blackbirds or occasionally some other synanthropic avian species.
